# The impact of depression and physical multimorbidity on health-related quality of life in China: a national longitudinal quantile regression study

**DOI:** 10.1038/s41598-022-25092-7

**Published:** 2022-12-14

**Authors:** Tianxin Pan, Kanya Anindya, Nancy Devlin, Stewart W. Mercer, Barbara McPake, Alex van Heusden, Yang Zhao, Xiuqi Hao, Tiara Marthias, John Tayu Lee

**Affiliations:** 1 Melbourne School of Population and Global Health, The University of Melbourne, 207 Bouverie Street, Melbourne, Australia; 2 School of Public Health and Community Medicine, University of Gothenburg, Sahlgrenska Academy, Gothenburg, Sweden; 3 Usher Institute of Population Health Sciences and Informatics, College of Medicine and Veterinary Medicine, University of Edinburgh, Edinburgh, UK; 4 The George Institute for Global Health, University of New South Wales, Sydney, Australia; 5 The George Institute for Global Health, Beijing, China; 6 Peking Union Medical College Hospital, Chinese Academy of Medical Sciences, Beijing, China; 7 College of Health and Medicine, Australian National University, Canberra, Australia; 8 Department of Primary Care and Public Health, School of Public Health, Imperial College, London, UK; 9 Department of Global Health and Population, Harvard T. H Chen School of Public Health, Harvard University, Boston, US

**Keywords:** Geriatrics, Public health, Quality of life

## Abstract

The co-occurrence of mental and physical chronic conditions is a growing concern and a largely unaddressed challenge in low-and-middle-income countries. This study aimed to investigate the independent and multiplicative effects of depression and physical chronic conditions on health-related quality of life (HRQoL) in China, and how it varies by age and gender. We used two waves of the China Health and Retirement Longitudinal Study (2011, 2015), including 9227 participants aged ≥ 45 years, 12 physical chronic conditions and depressive symptoms. We used mixed-effects linear regression to assess the effects of depression and physical multimorbidity on HRQoL, which was measured using a proxy measure of Physical Component Scores (PCS) and Mental Component Scores (MCS) of the matched SF-36 measure. We found that each increased number of physical chronic conditions, and the presence of depression were independently associated with lower proxy PCS and MCS scores. There were multiplicative effects of depression and physical chronic conditions on PCS (− 0.83 points, 95% CI − 1.06, − 0.60) and MCS scores (− 0.50 points, 95% CI − 0.73, − 0.27). The results showed that HRQoL decreased markedly with multimorbidity and was exacerbated by the presence of co-existing physical and mental chronic conditions.

## Introduction

The prevalence of multimorbidity, defined as having two or more co-existing mental or physical chronic conditions, is rising rapidly in low-and middle-income countries (LMICs)^1,2^. A recent study in China found that the prevalence of physical multimorbidity was 62% among middle-aged and elderly in China in 2015^3^. Findings from the China Mental Health Survey suggested that the prevalence of mental disorders was 9.3% in 2013 among Chinese adults, and most mental disorders have become more common over the past 30 years^4^. The rising prevalence of physical and mental chronic conditions is expected to pose significant challenges to the health system in China^3,5^.

Multimorbidity can involve different patterns of physical and mental chronic conditions, combinations, or clusters. To date, evidence suggests that the association between physical and mental chronic conditions can be bi-directional and the link between physical and mental chronic conditions might go in both directions^6^. A study in Scotland found that the prevalence of multimorbidity was 23.0% for those aged between 45 and 54 years and 76.1% for those aged 75 years above in 2007. However, among patients with multimorbidity, the presence of both physical and mental chronic conditions (i.e. mental-physical multimorbidity) was the most common multimorbidity pattern (approximately 50% of those with multimorbidity) in the non-elderly adult population^7^. A more recent study in Singapore found that the prevalence of multimorbidity was 39.2% in those aged between 45 and 64 years in 2016, but the prevalence of mental-physical multimorbidity was only 3.3%^8^. Previous studies have shown that the adverse impact of physical chronic conditions on individuals can be further exacerbated by the co-existence of mental chronic conditions, as the latter raises the complexities of clinical treatment and patient management^9–13^.

Health-related Quality of Life (HRQoL) is an outcome measure that is commonly used in clinical settings and population health surveys. Several studies have shown that multimorbidity has negative effects on HRQoL^14–16^. However there are limited evidence on the multiplicative (synergistic) effects of mental and physical chronic conditions, and they were all from high-income countries^17–20^. There is a paucity of research investigating the independent and multiplicative effects of mental and physical multimorbidity on HRQoL in a LMIC setting, where the prevalence and pattern of multimorbidity, and the ability of its health care system and social support service in mitigating the potential combined impact of physical illness and mental disorder on health outcomes might be different. Furthermore, existing research tends to focus on average HRQoL, ignoring the fact that the impact of multimorbidity may differ for individuals with varying levels of HRQoL.

To fill this critical knowledge gap, in this study, we used nationally representative panel data from China to investigate the independent and multiplicative effects of depression and physical multimorbidity on HRQoL in China, and aim to understand how the multiplicative effects vary by population segments (i.e., across age, by sex, and among people with poorer and better health).

## Method

### Sample and data

We used data from the China Health and Retirement Longitudinal Study (CHARLS), which collects information on individuals aged 45 years or older in China and their household characteristics. The survey data included measures of physical and psychological health, demographics, and socio-economic status (SES) information^21^.

We utilised two waves (2011 and 2015) of the CHARLS data that are publicly available. The baseline sample comprised of 17,708 individuals, of which 13,702 also had measurements on blood pressures. There were 10,063 individuals who remained in the 2015 wave, among which, 9657 had HRQoL measured in both waves. After removing individuals with missing values for key independent variables (4.4%), our final sample consisted of 9227 individuals each year, thus a total of 18,454 pooled sample observations in the panel data of two survey waves.

### Variables

A total of 12 physical chronic conditions were used to measure physical multimorbidity, including diagnosed hypertension and 11 self-reported diagnosed physical chronic conditions (diabetes, dyslipidaemia, heart disease, stroke, cancer, chronic lung disease, digestive disease, liver disease, kidney disease, arthritis and asthma). Individuals were defined as hypertensive if they had either a systolic blood pressure (SBP) ≥ 140 mmHg, a diastolic blood pressure (DBP) ≥ 90 mmHg, or if they were taking anti-hypertension medicines at the time of the survey^22^. We counted the number of physical chronic conditions reported for each participant to identify those with physical multimorbidity. We used the presence of depressive symptoms as an indicator of the presence of a mental health condition^13,23^. Depressive symptoms were identified using a self-reported Center for Epidemiologic Studies Depression 10 items (CES-D10) score, which had been identified as a valid, reliable and useful mental health assessment tool for elderly in China^24^. An individual with a score greater than ten was identified as having depression^25,26^. Respondents who had both depressive symptoms and physical chronic conditions were defined as having co-existing depression and physical multimorbidity.

The main HRQoL outcomes included the proxy physical component score (PCS) and mental component score (MCS) from the matched Short Form 36 (SF-36) constructed based on CHARLS questionnaires^27,28^. Both PCS and MCS range in values from 0 to 100, with 0 score indicating the lowest level of functioning and 100 score indicating the highest possible level of functioning. SF-36 is the most widely used HRQoL measure of studies on multimorbidity and HRQoL^14^. Standard SF-36 is a 36-question comprehensive health survey that covers eight domains used to define quality of life, including physical functioning, bodily pain, role physical, general health, mental health, social functioning, vitality and role emotional. The CHARLS questionnaire does not include any standard HRQoL instruments but contains a wide range of health indicators that can be used to construct the SF-36 based measure. We used the proxy measure of SF-36 including summarised PCS and MCS constructed by Hao et al^27,28^. Items and variables used to construct the proxy SF-36 measure are provided in the appendix (Figure S1). Details on the construction of the proxy measure and psychometric assessment has been described elsewhere^27,28^.

The following variables were included as covariates: year, age, gender, marital status (married and partnered, and otherwise), residency (rural or urban), Hukou (household registration system) status (agricultural or rural Hukou or non-agricultural or urban Hukou), geographical region (four regional classes), education (illiterate, primary school, secondary school, college and above), socio-economic status quartiles (annual per capita household consumption expenditure, Q1 being the poorest and Q4 being the richest), work type (formally employed, farming, self-employed and family business, and unemployed or retired)^26^ and disability (reported difficulties in activity of daily living)^13^.

### Statistical analyses

We described the prevalence of physical multimorbidity, depression, and co-existing of depression and physical multimorbidity across different population subgroups in two waves. We investigated socio-demographic correlates of multimorbidity using a mixed-effect logistic regression model.

We summarised the mean of outcomes by the number of physical chronic conditions, the presence of depression, and across different population subgroups. We examined the multiplicative effect of depression and physical multimorbidity on HRQoL using a mixed-effects linear regression model. Our null hypothesis was that the effect of having depression and physical chronic conditions on HRQoL was simply additive, no more than the sum of each condition. We tested the hypothesis by including two-way interaction terms between the number of physical chronic conditions and depression in the regression models. A statistically significant and negative coefficient for the interaction terms (negative coefficients in linear regressions of HRQoL) suggests that the combined adverse effect of two conditions was more than the additive effect of each one of them independently.

We examined how the effects of depression and physical multimorbidity on HRQoL vary by population segments through subgroup analysis and quantile analysis. We examined whether the association between physical chronic conditions, depression and HRQoL may be different for people at different levels of quality of life. In this analysis, we ran a series of quantile regression models at the 25th, 50th, 75th percentiles of the HRQoL outcomes. Quantile regression fit a line to minimizes the sum of absolute residuals. The objective is to estimate the median (as well as 25th and 75th percentile) of the outcome variable conditional on independent variables. Quantile regression analysis has been increasingly adopted in health systems research^29,30^. The method is robust to outliers because it allows for studying the full distribution of the outcome variable and is suitable for modelling outcomes such as HRQoL, which are often skewed or not normally distributed. The coefficients at lower quartile (i.e., the 25th percentiles) present the association between multimorbidity and outcomes on those with low HRQoL, while higher quartile (i.e. the 75th) reflects the association for those with higher HRQoL. We further examined the effect of number of chronic conditions on HRQoL to explore the burden of multimorbidity on HRQoL as an additional analysis. We reported the regression coefficient with 95% confidence intervals (CI). All statistical analyses were conducted using STATA 15.0. All methods were carried out in accordance with the Declaration of Helsinki.

### Ethical approval and consent to participate

The Biomedical Ethics Review Committee of Peking University approved the CHARLS study (approval number: IRB00001052–11015). Informed consent was obtained from participants.

## Results

### Prevalence of depression and physical multimorbidity

We analysed data from 9227 respondents. The median age of the participants was 58 years (inter-quartile range [IQR] 51–64) in 2011 and 62 years (IQR 55–68) in 2015. The majority of the participants were married, lived in a rural area, illiterate, and worked in the agricultural sector (Appendix Table S1). As shown in Table 1, the prevalence of multimorbidity was 55.8% among Chinese aged over 45 years in 2011, which increased to 66.7% to 2015. In 2015, the prevalence of depression and physical multimorbidity was 30.6% and was higher among older ages and females (female vs male: 37.3% and 23.4%).

**Table 1:** Prevalence of multimorbidity among Chinese adults aged 45 years and above.

	2011				2015			
	Depression	Any type of multimorbidity	Physical multimorbidity	Mental-Physical multimorbidity	Depression	Any type of multimorbidity	Physical multimorbidity	Mental-Physical multimorbidity
	% (95% CI)	% (95% CI)	% (95% CI)	% (95% CI)	% (95% CI)	% (95% CI)	% (95% CI)	% (95% CI)
Full sample	35.4 (33.4, 37.5)	55.8 (54.0, 57.6)	25.7 (24.3, 27.1)	30.1 (28.3, 31.9)	33.9 (32.1, 35.7)	66.7 (65.1, 68.2)	36.1 (34.7, 37.5)	30.6 (28.9, 32.4)
**Age group**								
Age 45–54	31.3 (28.3, 34.5)	44.3 (40.6, 48.1)	19.7 (17.7, 21.8)	24.7 (22.2, 27.3)	30.5 (27.3, 33.8)	55.9 (53.0, 58.8)	29.8 (27.4, 32.3)	26.1 (23.3, 29.1)
Age 55–64	36.8 (34.4, 39.3)	59.7 (57.5, 61.9)	27.7 (25.7, 29.9)	32.0 (29.8, 34.3)	33.0 (30.7, 35.4)	65.4 (63.0, 67.8)	35.8 (33.9, 37.7)	29.6 (27.4, 31.9)
Age 65–74	39.2 (36.1, 42.4)	67.3 (63.8, 70.6)	32.1 (29.0, 35.4)	35.2 (32.2, 38.3)	38.4 (35.7, 41.2)	75.6 (73.3, 77.7)	39.4 (36.9, 41.9)	36.2 (33.5, 39.0)
Age 75 +	39.4 (33.7, 45.5)	64.2 (59.1, 69.1)	29.2 (24.5, 34.3)	35.1 (29.8, 40.8)	32.9 (28.9, 37.1)	72.1 (68.5, 75.4)	41.8 (37.4, 46.6)	30.3 (26.5, 34.3)
**Gender**								
Male	27.9 (25.7, 30.3)	51.2 (49.2, 53.3)	28.2 (26.4, 30.2)	23.0 (21.0, 25.1)	26.0 (24.0, 28.0)	63.2 (61.2, 65.1)	39.8 (38.0, 41.6)	23.4 (21.5, 25.4)
Female	42.2 (40.0, 44.5)	60.0 (57.6, 62.3)	23.4 (21.6, 25.3)	36.6 (34.5, 38.8)	41.1 (39.1, 43.2)	70.0 (68.1, 71.8)	32.7 (30.9, 34.5)	37.3 (35.4, 39.3)
**Marital status**								
Unmarried	48.1 (44.6, 51.5)	64.1 (60.4, 67.7)	22.7 (19.8, 25.8)	41.4 (38.0, 44.9)	44.8 (41.5, 48.1)	72.6 (69.7, 75.4)	32.3 (29.3, 35.5)	40.3 (37.0, 43.6)
Married	33.6 (31.5, 35.7)	54.6 (52.7, 56.5)	26.1 (247, 27.6)	28.5 (26.6, 30.4)	31.9 (30.0, 33.8)	65.6 (63.9, 67.4)	36.8 (35.3, 38.2)	28.9 (27.1, 30.7)
**Residency**								
Urban	24.5 (21.3, 27.9)	58.5 (54.6, 62.3)	37.3 (33.9, 40.9)	21.2 (18.3, 24.3)	21.7 (18.7, 25.0)	66.8 (63.7, 69.8)	47.1 (44.3, 50.0)	19.7 (16.9, 22.9)
Rural	41.4 (39.1, 43.7)	56.7 (54.6, 58.9)	21.8 (20.4, 23.3)	34.9 (32.8, 37.2)	39.7 (37.7, 41.6)	67.8 (65.8, 69.7)	31.7 (30.1, 33.3)	36.1 (34.2, 38.1)
Migrant	29.8 (25.7, 34.3)	51.1 (45.6, 56.6)	25.4 (22.1, 29.0)	25.7 (22.0, 29.8)	30.2 (26.5, 34.2)	64.1 (59.6, 68.3)	37.4 (34.5, 40.4)	26.7 (23.4, 30.3)
**Region**								
East China	26.6 (23.4, 30.1)	47.8 (44.6, 51.0)	26.3 923. 7, 29.1)	21.5 (18.9, 24.3)	25.3 (22.7, 28.1)	58.7 (55.9, 61.4)	36.3 (34.0, 38.6)	22.4 (20.2, 24.8)
Middle China	39.1 (36.1, 42.2)	58.6 (55.9, 61.3)	25.7 (23.5, 28.0)	32.9 (30.1, 35.9)	37.7 (34.9, 40.5)	70.4 (68.1, 72.6)	35.9 (33.5, 38.3)	34.6 (31.7, 37.5)
West China	42.4 (39.2, 45.8)	61.5 (58.7, 64.3)	24.2 (22.0, 26.5)	37.3 (34.3, 40.5)	41.6 (38.8, 44.6)	72.2 (69.8, 74.4)	34.4 (31.9, 36.9)	37.8 (35.0, 40.7)
Northeast China	31.0 (25.7, 36.9)	56.4 (50.8, 61.9)	29.1 (24.7, 33.9)	27.4 (224, 32.9)	25.3 (20.6, 30.7)	65.3 (60.1, 70.2)	42.4 (36.7, 48.3)	22.9 (18.6, 27.8)
**Educational level**								
Illiterate	44.4 (41.9, 46.9)	60.4 (58.1, 62.6)	22.0 (20.3, 23.8)	38.3 (36.0, 40.7)	43.4 (41.2, 45.7)	71.0 (69.0, 73.0)	31.4 (29.6, 33.3)	39.6 (37.4, 41.9)
Primary	34.2 (31.4, 37.0)	58.7 (55.6, 61.8)	29.5 (26.4, 32.8)	29.3 (26.6, 32.0)	33.0 (30.6, 35.5)	69.4 (66.6, 72.1)	39.2 (36.3, 42.3)	30.2 (27.8, 32.7)
Secondary	28.3 (25.9, 30.9)	49.4 (46.4, 52.5)	26.2 (23.4, 29.3)	23.2 (21.1, 25.5)	24.5 (22.3, 26.8)	60.9 (58.3, 63.5)	39.5 (36.9, 42.2)	21.4 (19.3, 23.6)
Tertiary	17.9 (14.4, 22.0)	45.2 (41.3, 49.1)	31.0 (27.8, 34.3)	14.2 (11.3, 17.6)	17.8 (14.6, 21.5)	56.4 (51.9, 60.8)	40.6 (37.4, 44.)	15.7 (12.8, 19.2)
**HH consumption quantile**								
Q1 (poorest)	39.7 (36.4, 43.2)	54.1 (50.3, 57.8)	21.0 (18.9, 23.3)	33.0 (30.0, 36.3)	36.9 (34.5, 39.4)	67.0 (64.6, 69.4)	34.0 (31.9, 36.1)	33.1 (30.7, 35.6)
Q2	38.8 (35.9, 41.7)	58.1 (55.4, 60.7)	24.9 (22.9, 27.0)	33.2 (30.5, 36.0)	35.7 (33.0, 38.4)	66.4 (63.9, 68.8)	34.1 (31.7, 36.5)	32.4 (29.8, 35.0)
Q3	37.6 (34.7, 40.5)	56.0 (53.2, 58.7)	24.1 (21.6, 26.7)	31.9 (29.4, 34.5)	33.4 (30.4, 36.4)	66.5 (62.9, 70.0)	36.5 (33.6, 39.5)	30.0 (27.3, 32.9)
Q4 (richest)	27.5 (24.9, 30.3)	55.2 (52.4, 58.1)	31.5 (28.9, 34.2)	23.8 (21.5, 26.2)	30.1 (27.4, 33.0)	66.9 (64.4, 69.3)	39.3 (36.6, 42.0)	27.6 (25.1, 30.3)
**Work Type**								
Formally employed	17.6 (14.66, 23.4)	36.2 (31.7, 40.9)	22.3 (19.7, 25.1)	13.9 (10.7, 17.7)	20.6 (16.9, 24.8)	50.5 (45.7, 55.3)	32.6 (29.6, 25.8)	17.9 (14.5, 21.7)
Self-employed	25.6 (22.1, 29.5)	45.3 (40.5, 50.2)	25.5 (21.6, 29.8)	19.8 (16.7, 23.3)	27.7 (23.3, 32.6)	64.0 (59.1, 68.6)	39.1 (33.3, 45.2)	24.9 (20.9, 29.5)
Farming	40.6 (38.2, 42.9)	57.0 (55.0, 59.0)	22.7 (21.2, 24.2)	34.4 (32.2, 36.6)	38.5 (36.4, 40.7)	67.4 (65.4, 69.4)	32.9, (31.0, 34.9)	34.5 (32.5, 36.5)
Unemployed/retired	36.5 (33.8, 39.3)	64.7 (62.2, 67.1)	32.2 (29.8, 34.7)	32.5 (29.9, 35.2)	34.7 (32.3, 37.2)	72.6 (70.7, 74.5)	40.5 (38.3, 42.7)	32.3 (29.8, 34.6)
**Functional limitation**								
No	34.0 (30.8,37.3)	17.7 (15.4, 20.2)	78.5 (75.9, 80.8)	39.2 (36.0, 42.5)	40.1 (37.5, 42.8)	14.7 (12.5, 17.2)	71.4 (68.6, 74.1)	43.2 (40.5, 46.0)
Yes	66.0 (62.7, 69.2)	82.3 (79.8, 84.6)	21.5 (19.2, 24.1)	60.8 (57.5, 64.0)	59.9 (57.2, 62.5)	85.3 (82.8, 87.5)	28.6 (25.9, 31.4)	56.8 (54.0, 59.5)

CI, confidence interval. Percentage is based on weighted sample. N = 9227. Percentages may not total 100 because of rounding.

Table 2 presents the factors associated with depression, any type of multimorbidity and mental-physical multimorbidity. Respondents who were female, not married, having a non-agricultural Hukou, not residing in East China, illiterate, unemployed or worked in the farming sector, and having difficulties in activity of daily living were more likely to have depression, compared to their counterparts. The probability of having any type of multimorbidity was higher among people who were female, living in regions other than East China, having higher household consumption expenditure, unemployed or retired, and having difficulties in activity of daily living. The probability of having depression and physical multimorbidity was higher in respondents aged between 65 and 74 years compared to aged 45 and 54 years (adjusted odds ratio [aOR] 1.32, 95%CI 1.13, 1.54), among female (aOR 2.15, 95% CI 1.91, 2.42), having a rural Hukou (aOR 2.26, 95% CI 1.81, 2.83), unemployed or retired (aOR 1.75, 95%CI 1.45, 2.11) and illiterate compared to their counterparts.

**Table 2:** Factor associated with depression, any type of multimorbidity, mental-physical multimorbidity.

	Depression			Any multimorbidity			Mental-physical multimorbidity		
	aOR	95% CI	*p* value	aOR	95% CI	*p* value	aOR	95% CI	*p *value
**Age group**									
Age 55–64	1.03	(0.92, 1.16)	0.571	3.38	(2.81, 4.07)	< 0.001	1.18	(1.04, 1.34)	0.011
Age 65–74	1.05	(0.91, 1.20)	0.531	8.90	(6.93, 11.42)	< 0.001	1.32	(1.13, 1.54)	< 0.001
Age 75 +	0.77	(0.62, 0.95)	0.017	10.01	(6.91, 14.51)	< 0.001	0.99	(0.79, 1.25)	0.936
**Gender**									
Female	2.00	(1.79, 2.23)	< 0.001	2.47	(2.03, 3.00)	< 0.001	2.15	(1.91, 2.42)	< 0.001
Marital status									
Married	0.58	(0.50, 0.67)	< 0.001	0.78	(0.61, 1.01)	0.057	0.61	(0.53, 0.72)	< 0.001
**Residency**									
Rural	2.24	(1.82, 2.75)	< 0.001	1.24	(0.91, 1.70)	0.180	2.26	(1.81, 2.83)	< 0.001
Migrant	1.48	(1.20, 1.81)	< 0.001	1.29	(0.95, 1.76)	0.106	1.51	(1.21, 1.88)	< 0.001
**Region**									
Middle China	2.19	(1.65, 2.92)	< 0.001	3.66	(2.15, 6.22)	< 0.001	2.35	(1.65, 3.37)	< 0.001
West China	2.50	(1.90, 3.28)	< 0.001	5.18	(3.12, 8.61)	< 0.001	2.87	(2.05, 4.02)	< 0.001
Northeast China	1.38	(0.92, 2.06)	0.118	2.53	(1.21, 5.29)	0.014	1.55	(0.95, 2.53)	0.079
**Educational level**									
Primary	0.75	(0.66, 0.85)	< 0.001	1.05	(0.83, 1.33)	0.697	0.76	(0.66, 0.88)	< 0.001
Secondary	0.57	(0.49, 0.66)	< 0.001	0.71	(0.55, 0.92)	0.009	0.54	(0.46, 0.63)	< 0.001
Tertiary	0.43	(0.34, 0.52)	< 0.001	0.52	(0.37, 0.73)	< 0.001	0.39	(0.31, 0.50)	< 0.001
**Household consumption quantile**									
Q2	1.01	(0.90, 1.14)	0.806	1.40	(1.18, 1.67)	< 0.001	1.07	(0.95, 1.22)	0.269
Q3	1.05	(0.93, 1.18)	0.440	1.28	(1.07, 1.53)	0.006	1.10	(0.96, 1.25)	0.167
Q4 (richest)	0.98	(0.86, 1.11)	0.726	1.43	(1.19, 1.73)	< 0.001	1.08	(0.94, 1.24)	0.292
**Work type**									
Self-employed	1.10	(0.87, 1.38)	0.418	0.98	(0.72, 1.35)	0.921	1.06	(0.83, 1.37)	0.632
Farming	1.45	(1.24, 1.71)	< 0.001	1.04	(0.83, 1.30)	0.727	1.40	(1.17, 1.67)	< 0.001
Unemployed/retired	1.69	(1.42, 2.00)	< 0.001	1.72	(1.35, 2.19)	< 0.001	1.75	(1.45, 2.11)	< 0.001
**Functional limitation**									
Reported functional limitation	4.45	(3.97, 4.99)	< 0.001	6.15	(5.02, 7.53)	< 0.001	4.76	(4.23, 5.37)	< 0.001

*AOR* Adjusted odds ratio, *CI* confidence interval. Mixed-effect multivariate logistic regression models are used to estimate the associations of socio-demographic factors with depression, any type of multimorbidity, mental-physical multimorbidity, respectively. Models were adjusted for age, sex, marital status, residency, Hukou status, geographical region, education, socio-economic status quartiles, work type and disability.

### Depression and physical multimorbidity and health-related quality of life

The unadjusted mean of the two HRQoL outcomes by type of multimorbidity and by demographic and socio-economic groups are reported in Table S2. The adjusted effects on physical and mental chronic conditions on HRQoL are presented in Table 3. We found that respondents with more physical conditions had lower PCS and MCS. An increase of one in the number of physical conditions led to a statistically significant reduction of 2.27 (95% CI − 2.44, − 2.11) points in PCS and reduction of 0.38 (95% CI − 0.55, − 0.22) points in MCS, after adjusting for socio-economic and demographic factors. Independently, depression was also significantly associated with lower PCS (a reduction of 6.58, 95% CI − 7.19, − 5.97) and MCS (a reduction of − 25.26, 95% CI − 25.88, − 24.64).

**Table 3:** The effect of multimorbidity on proxy PCS and MCS score.

Variable	Overall^a^	25th percentile^b^	50th percentile^b^	75th percentile^b^
	Coef (95% CI)	Coef (95% CI)	Coef (95% CI)	Coef (95% CI)
**Proxy PCS score**				
Number of physical conditions	− 2.27***	− 2.11***	− 2.17***	− 2.19***
	(− 2.44, − 2.11)	(− 2.24, − 1.98)	(− 2.19, − 2.16)	(− 2.21, − 2.18)
Depression	− 6.58***	− 5.40***	− 6.04***	− 6.25***
	(− 7.19, − 5.97)	(− 6.89, − 3.91)	(− 6.18, − 5.90)	(− 6.34, − 6.16)
Physical conditions * Depression	− 0.83***	− 1.35***	− 1.59***	− 1.53***
	(− 1.06, − 0.60)	(− 1.66, − 1.03)	(− 1.62, − 1.56)	(− 1.55, − 1.51)
**Proxy MCS score**				
Number of physical conditions	− 0.38***	− 0.78***	− 0.31***	− 0.38***
	(− 0.55, − 0.22)	(− 0.96, − 0.61)	(− 0.34, − 0.28)	(− 0.40, − 0.36)
Depression	− 25.26***	− 23.36***	− 24.65***	− 24.68***
	(− 25.88, − 24.64)	(− 23.84, − 22.89)	(− 24.93, − 24.38)	(− 25.07, − 24.29)
Physical conditions * Depression	− 0.50***	− 0.09*	− 0.45***	− 0.38***
	(− 0.73, − 0.27)	(− 0.19, 0.01)	(− 0.49, − 0.41)	(− 0.40, − 0.36)

*CI* confidence interval, *PCS* Physical Component Scores, *MCS* Mental Component Scores. All estimates adjusted with sample weight. Models were adjusted for age, sex, marital status, residency, Hukou status, geographical region, education, socio-economic status quartiles, work type and disability.

^a^Coefficients were estimated using multilevel mixed-effect model.

^b^Coefficients were estimated using quantile regression analysis.

***p < 0.01, *p < 0.1.

There were significant interaction effects of depression and physical chronic condition on PCS (regression coefficient for interaction term = − 0.83, 95% CI − 1.06, − 0.60) and a lesser extent with MCS (regression coefficient for interaction term = − 0.50, 95% CI − 0.73, − 0.27). The results suggest that the impact of depression and physical multimorbidity was multiplicative rather than a simple additive effect on decreasing HRQoL.

### Quantile regression analysis and subgroup analysis

Table 3 also shows the differential impacts of depression and physical multimorbidity on the PCS and MCS at the 25th, 50th and 75th percentiles, after adjusting for covariates. Our findings suggest that the magnitude of the multiplicative effect of depression and physical multimorbidity on PCS and MCS was larger in the lower percentile (i.e. 25th) of the HRQoL distribution compared with the higher percentile (75th) of the HRQoL distribution. The full regression results are reported in Appendix (Tables S3 and S4).

**Figure 1:**
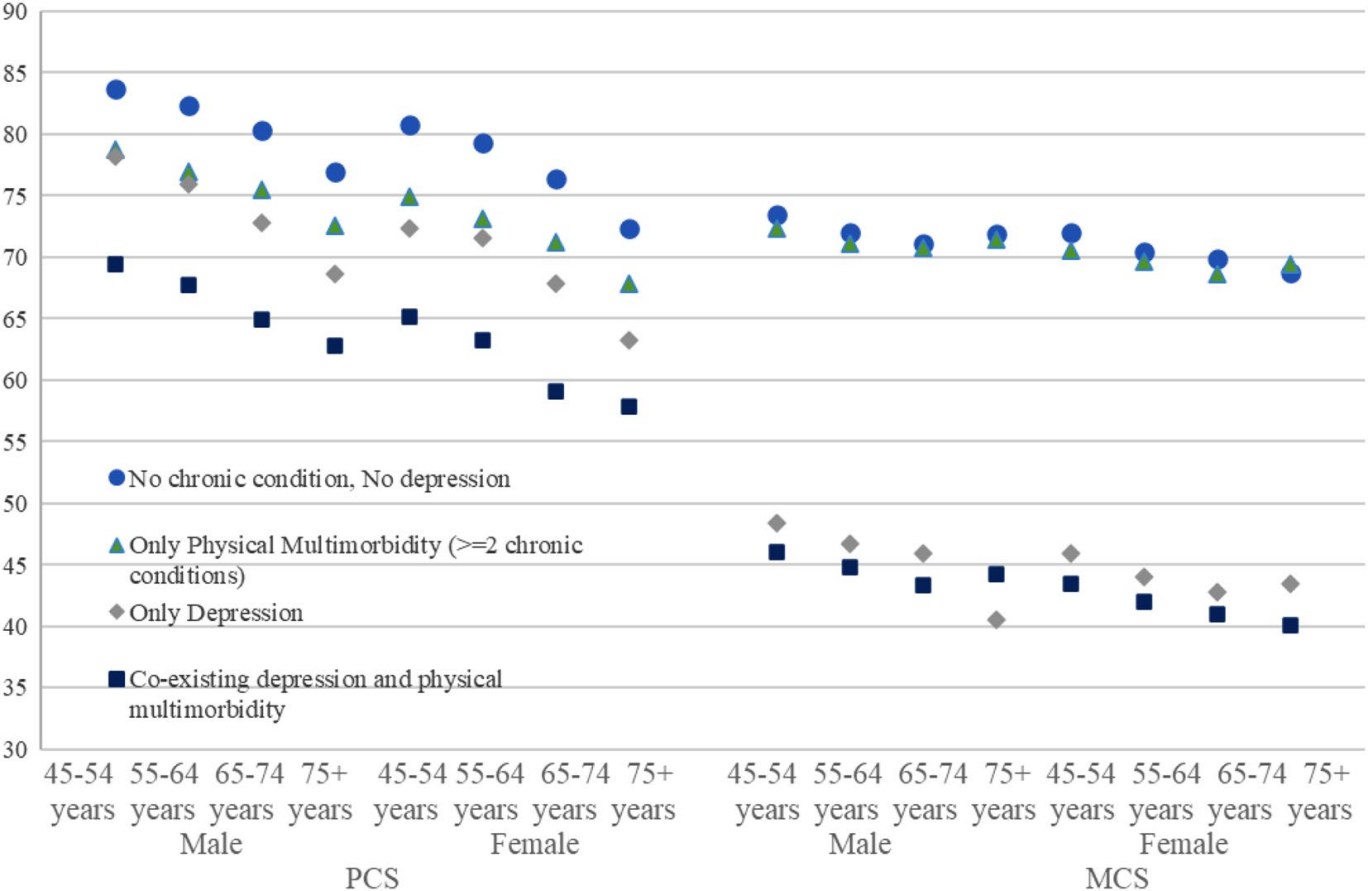
Estimated mean proxy Physical Component Scores (PCS) and Mental Component Scores (MCS) scores by age groups and sex. China Health and Retirement Longitudinal Study, 2011–2015. Mixed-effect linear models are used to estimate the association between multimorbidity and PCS and MCS scores. Models were adjusted for age, sex, marital status, residency, Hukou status, geographical region, education, socio-economic status quartiles, work type and disability.

Figure 1 shows the estimated mean PCS and MCS by type of multimorbidity across age and gender. Both PCS and MCS decrease with ages. PCS scores were on average five points lower for females compared to males within the same age group whereas the differences in MCS among males and females were smaller. For example, among those who were between 45 and 54 years, PCS decreased from 83.7 for males and 80.8 for females without any physical chronic conditions and depression to 69.4 for males and 65.1 for females with more than two physical chronic conditions and depression. In the same age group, MCS decreased from 73.5 for males and 72.0 for females without any condition to 46.1 and 44.2 among those who had more than two physical chronic conditions and depression.

We additionally estimated the mean proxy PCS and MCS by Hukou status and by educational level. Respondents with an agricultural (i.e. rural) Hukou had the lowest PCS and MCS compared to non-agricultural Hukou and migrants. Respondents with low education had lower PCS and MCS than those with high education level. The results are presented in Figure S2. In addition, we examined the effect of number of chronic conditions on HRQoL. An increased number of chronic conditions was associated with lower PCS and MCS scores. The results are reported in Table S5 and Figure S3 in the appendix.

## Discussion

Our results showed that in 2015 the prevalence of mental-physical multimorbidity was 30.6% among Chinese aged over 45 years. Increasing numbers of physical chronic conditions and depression were independently associated with lower PCS and MCS. There were significant interactions of depression and physical chronic condition, suggesting the effects on HRQoL was multiplicative rather than simply additive. The negative effects of co-occurring depression and physical multimorbidity on HRQoL were greatest in women and those in poorer health.

Our results from quantile regression analysis revealed a more detailed measure of the association between multimorbidity and HRQoL that was not able to find in existing studies using ordinary least squares regression or generalised linear model. Multimorbidity has significant effect on HRQoL at various point of the distribution, and it remain predictive even for those with high HRQoL.

Our findings align with previous studies in China and the international context, showing that physical chronic conditions and depression impact negatively on HRQoL, independently^14,31–34^. Our results further indicated that depression, compared to each increased number of physical chronic conditions, had a greater effect on lowering HRQoL^18,20^.

More importantly, our result showed that the multiplicative effects of co-existing of depression and physical multimorbidity on quality of life impairment exist in China, whereas previous evidence that from high-income settings (Spain, Singapore and US) found similar effects^17–20^. This suggests that the mechanism by which co-existing mental and physical multimorbidity have multiplicative deleterious effects on quality of life may be similar across populations in different settings. This is also in line with published studies showing that mental conditions can increase the level of health care use and medical cost in the presence of physical multimorbidity^9,35^.

Our study showed that a high proportion of the study population had multimorbidity, with a substantial percentage having both depression and physical conditions. The multiplicative negative effects of mental-physical multimorbidity on HRQoL indicates the challenges and complexity in managing the co-existing conditions. Health care systems that are typically organised around single diseases can no longer effectively address the burden from multimorbidity. It is crucial to priorities the integration of physical and mental health care to improve patients’ outcome. An example of this approach was implemented in the United Kingdom, where recently issued national guidelines provided clinical directives for early assessment and management of patients with multimorbidity^36^.

The health delivery system in China is still single disease focused, hospital-centred and volume-driven. Renewing the focus on health promotion and management of multimorbidity would require a strong, affordable, and integrated primary health care system. China has made remarkable progress on this front^37^. However, further strategies should be strengthened to address the multimorbidity challenges, including shifting from secondary care to more integrated primary care, which would allow for better generalist health services^38,39^. Also, considering that the negative multiplicative effects are more pronounced among people with poorer health, more attention should be given to these groups to deliver targeted and tailored care and prevention services^40,41^. Efficient management of mental and physical multimorbidity needs an integrated care model and implementation of interventions in different settings, including both clinical settings and community. A recent systematic review found little evidence of an effect of HRQoL interventions for people with multimorbidity in primary care and community settings^42,43^. The review suggested that future research should consider patient experience of care, optimising medicines management and targeted patient health behaviours such as exercise. More research that addresses the challenges of mental-physical multimorbidity is needed to inform the development of interventions that can be applied to the wider community in China.

We presented the first study that systematically assessed the multiplicative effects of depression and physical multimorbidity on HRQoL and investigated differential impacts on population segments in China using longitudinal data and a quantile regression approach. However, our study has several important caveats. The first relates to the use of the proxy SF-36 based on available survey questions rather than using the standard SF-36 instrument. To the best of our knowledge, there is no public national representative longitudinal dataset that have used a standard HRQoL instrument in China. Despite the possible bias using the constructed instrument, our results are consistent with the literature on the impact of deteriorating physical and mental health using the SF-36 instrument, suggesting the constructed measure has a degree of face validity. Our study estimated HRQoL through PCS and MCS, rather than health utilities^44^. This was because not all corresponding items required to calculate utilities based on SF-6D^45^ was available in CHARLS questionnaires. The second relates to the use of self-reported measures in physical chronic conditions. Self-reported measures of physical chronic conditions may underestimate their prevalence, particularly among those from lower SES and educational backgrounds and rural areas due to restricted access to healthcare services. Also, we did not consider different combinations of physical chronic conditions. Third, as CHARLS does not contain other measures on mental health conditions except for depression, our estimated effects of having depression may capture a wider effects of mental health conditions. In addition, depressive symptoms are a part of HRQOL measures, our models included conceptually similar variables on both sides of the equation^46^. These might explain the very large effects we found of depression on both MCS and PCS. However, it does not alter our conclusion that care for people with depression needs to be prioritised given its substantial impact on not only mental health but also physical health. Future research that considers different combinations of chronic conditions and include more mental conditions can enrich our understanding of the impact of mental and physical multimorbidity on HRQoL. Standard HRQoL instruments should be incorporated in future large-scale population health surveys in China.

In conclusion, the present study has shown that HRQoL decreased markedly with multimorbidity and is exacerbated by the presence of both mental and physical chronic conditions. There is a need to prioritise interventions to health outcomes for patients suffering from co-occurring depression and physical health conditions in China.

## Supplementary Information

Supplementary Information.

## Data Availability

The data that support the findings of this study are available from the China Health and Retirement Longitudinal Study (CHARLS) study (charls.pku.edu.cn) but restrictions apply to the availability of these data. Data are however available from the authors upon reasonable request and with permission of the CHARLS study team.

## Code availability

Stata Code for the analysis is available from the authors upon reasonable request.
