# Supplementary material for: The impact of depression and physical multimorbidity on health-related quality of life in China: a national longitudinal quantile regression study
Source: Sci Rep. 2022 Dec 14;12:21620. doi: 10.1038/s41598-022-25092-7 (PMC9750988; doi:10.1038/s41598-022-25092-7)

Supplementary appendix

Table S1. Respondents' characteristics

Variables	2011			2015		
	N	%	(95% CI)	N	%	(95% CI)
Full sample	9227	100		9227	100	
**Age group**						
Age 45-54	3363	36.3	(34.5–38.1)	2102	22.8	(21.4–24.1)
Age 55-64	3726	39.4	(37.7–41.1)	3696	39.3	(38.0–40.7)
Age 65-74	1674	18.4	(17.3–19.5)	2505	26.7	(25.4–28.1)
Age 75+	464	5.8	(5.1–6.7)	924	11.1	(10.2–12.2)
**Gender**						
Male	4394	47.8	(47.0–48.6)	4394	47.8	(47.0–48.6)
Female	4833	52.2	(51.4–53.0)	4833	52.2	(51.4–53.0)
**Marital status**						
Unmarried	1071	12.6	(11.5–13.7)	1363	15.5	(14.3–16.7)
Married	8156	87.4	(86.2–88.4)	7864	84.5	(83.3–85.7)
**Residency**						
Urban	1372	19.8	(16.4–23.7)	1404	19.9	(16.6–23.6)
Rural	5869	57.4	(52.0–62.6)	5805	56.8	(51.4–61.9)
Migrant	1986	22.7	(19.2–26.7)	2018	23.3	(19.9–27.1)
**Region**						
East China	2773	32.5	(27.2–38.2)	2773	32.5	(27.2–38.1)
Middle China	2793	29.3	(24.6–34.4)	2793	29.3	(24.6–34.5)
West China	2969	30.3	(25.6–35.3)	2969	30.3	(25.6–35.3)
Northeast China	692	8.0	(5.5–11.4)	692	8.0	(5.5–11.4)
**Educational level**						
Illiterate	4223	43.3	(40.9–45.8)	4223	43.3	(40.8–45.8)
Primary	2115	22.9	(21.5–24.3)	2115	22.9	(21.5–24.3)
Secondary	1953	22.3	(20.8–23.8)	1953	22.3	(20.8–23.9)
Tertiary	936	11.5	(9.9–13.2)	936	11.5	(9.9–13.2)
**Household consumption quantile**						
Q1 (poorest)	2294	23.4	(21.5–25.4)	2340	23.8	(22.0–25.6)
Q2	2307	22.9	(21.3–24.6)	2262	23.5	(22.0–25.0)
Q3	2311	24.5	(22.8–26.2)	2307	25.8	(24.4–27.2)
Q4 (richest)	2315	29.2	(26.3–32.1)	2318	26.9	(24.5–29.5)
**Work Type**						
Formally employed	1051	13.2	(11.0–15.8)	1179	13.9	(12.2–15.9)
Self-employed	644	6.9	(6.0–7.8)	615	7.0	(6.2–7.9)
Farming	5075	49.6	(45.8–53.3)	4343	42.6	(39.3–45.8)
Unemployed/retired	2457	30.3	(28.0–32.7)	3090	36.5	(34.4–38.6)
**Functional limitation**						
Yes	1417	0.1	(0.1–0. 2)	2028	0.2	(0.2–0.2)
No	7738	0.9	(0.8–0.8)	7199	0.8	(0.8–0.8)

Data Source: China Health and Retirement Longitudinal Study (2011, 2015)

Note: Ns are unweighted and percentages are weighted

Table S2. Statistic summary of health-related quality of life by mental and physical chronic conditions and socio-economic status

	N	PCS			MCS		
		mean	95% CI	P-value	mean	95% CI	P-value
Full sample	18454	74.5	(73.9 - 75.1)		61.9	(61.1- 62.7)	
No physical condition	3453	82.8	(82.2 - 83.4)	Ref.	67.0	(66.0- 67.9)	Ref.
1 physical condition	5063	78.6	(77.9- 79.3)	<0.0001	64.3	(63.3- 65.2)	<0.0001
>=2 physical condition	9938	69.5	(68.8- 70.1)	<0.0001	58.9	(58.0- 59.7)	<0.0001
Without depression	11724	79.8	(79.4- 80.2)	Ref.	72.2	(71.8- 72.6)	Ref.
Depression	6730	64.5	(63.8- 65.2)	<0.0001	42.5	(42.0- 43.1)	<0.0001
Male	8788	77.3	(76.7- 77.9)	Ref.	65.0	(64.3- 65.8)	Ref.
Female	9666	72.0	(71.3- 72.6)	<0.0001	59.0	(58.2- 59.9)	<0.0001
Urban	2776	78.0	(77.0- 79.0)	Ref.	68.8	(67.6- 70.0)	Ref.
Rural	11674	72.8	(72.0- 73.4)	<0.0001	59.1	(58.3- 60.0)	<0.0001
Migrant	4004	75.9	(74.7- 77.0)	0.009	62.8	(61.5- 64.1)	<0.0001
Illiterate	8446	71.0	(70.3- 71.7)	Ref.	57.2	(56.3- 58.1)	Ref.
Primary	4230	74.9	(74.0- 75.7)	<0.0001	62.2	(61.3- 63.2)	<0.0001
Secondary	3906	77.8	(77.0- 78.6)	<0.0001	65.7	(64.8- 66.6)	<0.0001
Tertiary	1872	80.6	(79.7- 81.4)	<0.0001	71.5	(70.1- 72.9)	<0.0001
Q1	4634	73.6	(72.8- 74.3)	Ref.	59.6	(58.6- 60.5)	Ref.
Q2	3569	74.1	(73.3- 74.8)	0.2068	60.7	(59.8- 61.6)	0.0334
Q3	4618	74.4	(73.7- 75.2)	0.0429	61.5	(60.5- 62.4)	0.0009
Q4	4633	75.8	(75.0- 76.5)	<0.0001	65.2	(64.2- 66.3)	<0.0001
No functional limitation	14937	78.3	(77.8- 78.7)	Ref.	64.7	(64.0- 65.4)	Ref.
Functional limitation	3445	57.1	(56.3- 57.9)	<0.0001	49.1	(48.1- 50.2)	<0.0001

Data Source: China Health and Retirement Longitudinal Study (2011 2015)

Note: All estimates adjusted with sample weight.

PCS: Physical Component Scores

MCS: Mental Component Scores

Table S3. The effect of multimorbidity on PCS score

Variable	Overall [a] Coef. (95% CI)	25th percentile [b] Coef. (95% CI)	50th percentile [b] Coef. (95% CI)	75th percentile [b] % change
**Number of physical conditions**	-2.27*** (-2.44 - -2.11)	-2.11*** (-2.24 - -1.98)	-2.17*** (-2.19 - -2.16)	-2.19*** (-2.21 - -2.18)
**Depression**	-6.58*** (-7.19 - -5.97)	-5.40*** (-6.89 - -3.91)	-6.04*** (-6.18 - -5.90)	-6.25*** (-6.34 - -6.16)
**Physcial conditions * Depression**	-0.83*** (-1.06 - -0.60)	-1.35*** (-1.66 - -1.03)	-1.59*** (-1.62 - -1.56)	-1.53*** (-1.55 - -1.51)
Year				
2015	0.98*** (0.67 - 1.30)	0.13*** (0.04 - 0.21)	0.28*** (0.26 - 0.29)	0.24*** (0.22 - 0.25)
Age group (ref: 45-54)				
age 55-64	0.56** (0.10 - 1.02)	1.30** (0.04 - 2.56)	0.67*** (0.64 - 0.70)	0.73*** (0.62 - 0.83)
age 65-74	0.06 (-0.52 - 0.63)	-0.07 (-0.59 - 0.44)	0.20*** (0.14 - 0.25)	0.01 (-0.05 - 0.08)
age 75+	-1.55*** (-2.40 - -0.69)	-0.26 (-2.16 - 1.64)	-1.50*** (-1.59 - -1.40)	-1.68*** (-1.75 - -1.61)
Gender (ref: male)				
Female	-1.97*** (-2.39 - -1.56)	-2.84*** (-4.82 - -0.86)	-1.74*** (-1.88 - -1.60)	-1.43*** (-1.46 - -1.40)
Marital status (ref: unmarried)				
Married	-0.59** (-1.17 - -0.01)	1.08 (-1.42 - 3.58)	-0.51*** (-0.69 - -0.33)	-0.79*** (-0.83 - -0.76)
Residency (ref:urban)				
Rural	-3.93*** (-4.67 - -3.19)	-1.25 (-5.35 - 2.85)	-2.97*** (-3.08 - -2.85)	-3.18*** (-3.22 - -3.15)
Migrant	-2.44*** (-3.18 - -1.70)	-0.44 (-3.36 - 2.48)	-1.95*** (-2.03 - -1.87)	-2.01*** (-2.09 - -1.93)
Region (ref:east China)				
Middle China	-1.55*** (-2.62 - -0.49)	-2.21*** (-3.60 - -0.82)	-1.35*** (-1.42 - -1.28)	-1.62*** (-1.74 - -1.50)
West China	-1.73*** (-2.74 - -0.72)	-2.62*** (-4.10 - -1.15)	-1.53*** (-1.58 - -1.49)	-1.63*** (-1.68 - -1.57)
Northeast China	-1.45* (-2.92 - 0.02)	-1.92*** (-3.09 - -0.75)	-1.10*** (-1.29 - -0.90)	-1.39*** (-1.44 - -1.34)
Education level (ref:illiterate)				
Primary	1.08*** (0.56 - 1.59)	1.00*** (0.28 - 1.72)	0.96*** (0.93 - 0.99)	1.10*** (1.01 - 1.19)
Secondary	1.93*** (1.37 - 2.50)	2.13*** (1.42 - 2.83)	2.20*** (2.16 - 2.24)	2.19*** (2.15 - 2.24)
Tertiary	2.35*** (1.59 - 3.11)	3.85** (0.91 - 6.79)	2.36*** (2.29 - 2.44)	2.34*** (2.15 - 2.53)
HH consumption per capita (ref:Q1)				
Q2	-0.33 (-0.81 - 0.15)	-1.19*** (-1.99 - -0.38)	-0.52*** (-0.59 - -0.45)	-0.50*** (-0.53 - -0.46)
Q3	-0.44* (-0.93 - 0.05)	-1.67*** (-2.42 - -0.93)	-0.91*** (-1.04 - -0.78)	-0.78*** (-0.81 - -0.74)
Q4 (richest)	-0.73*** (-1.24 - -0.22)	-0.15 (-1.25 - 0.94)	-0.66*** (-0.86 - -0.47)	-0.49*** (-0.60 - -0.37)
Work type				

Variable	Overall [a] Coef. (95% CI)	25th percentile [b] Coef. (95% CI)	50th percentile [b] Coef. (95% CI)	75th percentile [b] % change
Self-employed	-0.15	-1.22***	-0.26***	-0.11***
	(-0.99 - 0.70)	(-1.92 - -0.51)	(-0.37 - -0.16)	(-0.18 - -0.04)
Farming	-0.78**	-0.78***	-0.94***	-0.83***
	(-1.39 - -0.18)	(-1.32 - -0.25)	(-1.05 - -0.84)	(-0.85 - -0.80)
Unemployed /retired	-4.32***	-2.81***	-3.20***	-3.19***
	(-4.97 - -3.67)	(-4.27 - -1.36)	(-3.26 - -3.15)	(-3.27 - -3.11)
Functional limitation				
Reported functional limitation	-13.10***	-13.36***	-14.40***	-14.33***
	(-13.58 - -12.62)	(-14.56 - -12.15)	(-14.48 - -14.33)	(-14.40 - -14.27)

Data Source: China Health and Retirement Longitudinal Study (2011, 2015)

Note:

[a] Coefficients were estimated using multilevel mixed-effect model

[b] Coefficients were estimated using quantile regression analysis

PCS: Physical Component Scores

*** p<0.01, ** p<0.05, * p<0.1

Table S4. The effect of multimorbidity on MCS Score

Variable	Overall [a] Coef. (95% CI)	25th percentile [b] Coef. (95% CI)	50th percentile [b] Coef. (95% CI)	75th percentile [b] Coef. (95% CI)
**Number of physical conditions**	-0.38***	-0.78***	-0.31***	-0.38***
	(-0.55 - -0.22)	(-0.96 - -0.61)	(-0.34 - -0.28)	(-0.40 - -0.36)
**Depression**	-25.26***	-23.36***	-24.65***	-24.68***
	(-25.88 - -24.64)	(-23.84 - -22.89)	(-24.93 - -24.38)	(-25.07 - -24.29)
**Physical conditions * Depression**	-0.50***	-0.09*	-0.45***	-0.38***
	(-0.73 - -0.27)	(-0.19 - 0.01)	(-0.49 - -0.41)	(-0.40 - -0.36)
Year				
2015	0.71***	0.00	0.19***	0.17***
	(0.38 - 1.04)	(-0.05 - 0.06)	(0.14 - 0.25)	(0.14 - 0.20)
Age group (ref: 45-54)				
age 55-64	-0.43*	-0.44***	-0.19*	-0.45***
	(-0.90 - 0.03)	(-0.55 - -0.32)	(-0.38 - 0.00)	(-0.49 - -0.42)
age 65-74	-0.59**	-0.38***	-0.36***	-0.60***
	(-1.17 - -0.01)	(-0.57 - -0.18)	(-0.58 - -0.15)	(-0.67 - -0.52)
age 75+	-0.43	-3.26***	-0.40***	-0.27
	(-1.29 - 0.44)	(-4.76 - -1.76)	(-0.64 - -0.15)	(-0.73 - 0.20)
Gender (ref: male)				
Female	-0.93***	-1.37***	-0.74***	-0.53***
	(-1.34 - -0.51)	(-1.66 - -1.07)	(-0.87 - -0.61)	(-0.75 - -0.32)
Marital status (ref: unmarried)				
Married	0.17	-0.76*	0.22	-0.06***
	(-0.42 - 0.75)	(-1.64 - 0.13)	(-0.05 - 0.49)	(-0.10 - -0.01)
Residency (ref:urban)				
Rural	-3.06***	-2.62***	-3.01***	-2.76***
	(-3.84 - -2.28)	(-2.83 - -2.42)	(-3.34 - -2.68)	(-3.17 - -2.36)
Migrant	-1.99***	-3.33***	-2.38***	-2.17***
	(-2.76 - -1.23)	(-4.14 - -2.52)	(-2.63 - -2.12)	(-2.42 - -1.91)
Region (ref:east China)				
Middle China	-1.42*	-1.95***	-1.32***	-0.93***
	(-2.85 - 0.01)	(-2.24 - -1.67)	(-1.57 - -1.06)	(-1.02 - -0.85)
West China	-2.27***	-3.60***	-2.22***	-1.64***
	(-3.61 - -0.93)	(-4.17 - -3.02)	(-2.77 - -1.67)	(-1.71 - -1.57)
Northeast China	-1.06	-1.90***	-1.13***	-0.98***
	(-2.98 - 0.85)	(-2.41 - -1.40)	(-1.29 - -0.98)	(-1.27 - -0.69)
Education level (ref:illiterate)				
Primary	1.32***	2.63***	1.34***	1.31***
	(0.81 - 1.84)	(2.02 - 3.23)	(1.11 - 1.57)	(0.96 - 1.66)
Secondary	1.81***	3.29***	2.02***	1.80***
	(1.24 - 2.38)	(2.32 - 4.26)	(1.95 - 2.09)	(1.60 - 2.00)
Tertiary	3.74***	4.25***	3.85***	4.22***
	(2.98 - 4.51)	(3.86 - 4.64)	(3.69 - 4.00)	(3.87 - 4.56)
HH consumption per capita (ref:Q1)				
Q2	-0.26	-0.53	-0.31***	-0.37***
	(-0.75 - 0.23)	(-1.55 - 0.49)	(-0.41 - -0.21)	(-0.51 - -0.23)
Q3	0.18	-0.57***	0.41***	0.21**
	(-0.32 - 0.68)	(-0.90 - -0.25)	(0.21 - 0.61)	(0.02 - 0.39)
Q4 (richest)	0.55**	-0.70	0.71***	0.33
	(0.03 - 1.07)	(-1.81 - 0.41)	(0.54 - 0.89)	(-0.09 - 0.76)

Variable	Overall [a] Coef. (95% CI)	25th percentile [b] Coef. (95% CI)	50th percentile [b] Coef. (95% CI)	75th percentile [b] Coef. (95% CI)
Work type				
Self-employed	0.41	0.99***	0.95***	0.87***
	(-0.45 - 1.27)	(0.68 - 1.30)	(0.47 - 1.42)	(0.80 - 0.94)
Farming	-0.20	-0.89***	-0.35**	-0.48***
	(-0.81 - 0.42)	(-1.17 - -0.61)	(-0.63 - -0.06)	(-0.55 - -0.40)
Unemployed /retired	-0.12	-0.64***	0.31***	0.21
	(-0.79 - 0.54)	(-0.93 - -0.35)	(0.22 - 0.40)	(-0.14 - 0.57)
Functional limitation				
Reported functional limitation	-4.77***	-5.62***	-4.90***	-4.90***
	(-5.25 - -4.28)	(-5.89 - -5.34)	(-5.01 - -4.78)	(-5.22 - -4.59)

Data Source: China Health and Retirement Longitudinal Study (2011, 2015)

Note:

[a] Coefficients were estimated using multilevel mixed-effect model

[b] Coefficients were estimated using quantile regression analysis

MCS: Mental Component Scores

*** p<0.01, ** p<0.05, * p<0.1

Table S5. The effect of multimorbidity burden on HRQOL

Variable	PCS				MCS			
	Overall [a]	25th percentile [b]	50th percentile [b]	75th percentile [b]	Overall [a]	25th percentile [b]	50th percentile [b]	75th percentile [b]
	Coef (95% CI)	Coef (95% CI)	Coef (95% CI)	Coef (95% CI)	Coef (95% CI)	Coef (95% CI)	Coef (95% CI)	Coef (95% CI)
Overall (mean)	74.5	63.5	78.1	86.4	61.9	39.4	49.4	64.0
	(73.9, 75.0)	(63.1, 64.0)	(77.9, 78.3)	(86.3, 86.4)	(61.1, 62.7)	(48.5, 50.2)	(48.5, 50.2)	(63.4, 64.6)
Number of chronic conditions	-3.40***	-3.28***	-3.47***	-3.52***	-4.07***	-4.84***	-4.01***	-3.91***
	(-3.52 - -3.28)	(-3.45 - -3.11)	(-3.61 - -3.33)	(-3.53 - -3.52)	(-4.23 - -3.90)	(-5.10 - -4.59)	(-4.08 - -3.94)	(-3.92 - -3.91)
Year								
2015	1.46***	0.18*	0.36***	0.32***	2.86***	1.45***	0.59***	0.64***
	(1.14 - 1.78)	(-0.01 - 0.38)	(0.33 - 0.40)	(0.32 - 0.33)	(2.46 - 3.26)	(1.25 - 1.65)	(0.56 - 0.62)	(0.64 - 0.65)
Age group (ref: 45-54)								
age 55-64	0.80***	0.66**	-0.35	1.02***	0.45	1.83***	-0.22	0.18***
	(0.33 - 1.27)	(0.06 - 1.26)	(-0.87 - 0.16)	(0.99 - 1.04)	(-0.17 - 1.07)	(1.45 - 2.20)	(-0.54 - 0.10)	(0.15 - 0.20)
age 65-74	0.47	-0.76	-0.70*	0.66***	1.10***	2.02***	-0.23	1.02***
	(-0.11 - 1.06)	(-1.99 - 0.47)	(-1.46 - 0.06)	(0.63 - 0.69)	(0.32 - 1.89)	(1.52 - 2.52)	(-1.04 - 0.58)	(0.97 - 1.06)
age 75+	-0.96**	-3.68***	-2.91***	-0.46***	1.77***	2.89***	3.50***	2.83***
	(-1.83 - -0.08)	(-5.44 - -1.92)	(-4.22 - -1.60)	(-0.50 - -0.43)	(0.60 - 2.94)	(2.51 - 3.27)	(2.99 - 4.01)	(2.67 - 2.98)
Gender (ref: male)								
Female	-2.38***	-2.01***	-1.31*	-1.99***	-2.76***	-5.53***	-3.12***	-2.47***
	(-2.81 - -1.95)	(-2.34 - -1.67)	(-2.63 - 0.00)	(-2.01 - -1.97)	(-3.34 - -2.18)	(-6.34 - -4.71)	(-3.43 - -2.81)	(-2.53 - -2.41)
Marital status (ref: unmarried)								
Married	-0.14	-1.36**	2.46	-0.39***	2.11***	1.57***	2.45***	2.08***
	(-0.73 - 0.46)	(-2.45 - -0.26)	(-0.50 - 5.42)	(-0.44 - -0.34)	(1.31 - 2.90)	(1.29 - 1.85)	(2.15 - 2.76)	(2.01 - 2.16)
Residency(ref:urban)								
Rural	-4.50***	-4.03***	-3.14***	-3.81***	-5.69***	-0.19	-4.58***	-4.19***
	(-5.28 - -3.72)	(-5.20 - -2.87)	(-4.18 - -2.09)	(-3.83 - -3.79)	(-6.80 - -4.58)	(-1.18 - 0.79)	(-4.78 - -4.39)	(-4.27 - -4.11)
Migrant	-2.65***	-2.72***	-0.65	-2.39***	-2.95***	-0.68**	-2.61***	-2.66***
	(-3.42 - -1.87)	(-3.91 - -1.52)	(-3.31 - 2.02)	(-2.43 - -2.35)	(-4.02 - -1.89)	(-1.22 - -0.15)	(-2.71 - -2.52)	(-2.76 - -2.56)

**Region (ref:east China)**								
Middle China	-1.92***	-1.50***	-1.92***	-1.58***	-3.13***	-1.76***	-1.65***	-2.03***
	(-3.19 - -0.65)	(-1.76 - -1.25)	(-2.25 - -1.59)	(-1.59 - -1.57)	(-5.41 - -0.84)	(-2.79 - -0.73)	(-1.96 - -1.35)	(-2.09 - -1.98)
West China	-2.16***	-0.99	-2.87***	-1.69***	-4.21***	-2.98***	-2.46***	-3.51***
	(-3.36 - -0.97)	(-2.34 - 0.35)	(-5.00 - -0.74)	(-1.72 - -1.66)	(-6.34 - -2.09)	(-3.35 - -2.60)	(-3.13 - -1.80)	(-3.53 - -3.49)
Northeast China	-1.48*	-0.96**	-0.72***	-1.16***	-0.97	-1.47***	-0.40***	-0.28***
	(-3.21 - 0.24)	(-1.74 - -0.17)	(-0.95 - -0.48)	(-1.18 - -1.14)	(-4.00 - 2.07)	(-1.75 - -1.19)	(-0.58 - -0.22)	(-0.41 - -0.15)
**Education level (ref:illiterate)**								
Primary	1.32***	1.66***	-1.14	1.59***	2.28***	2.03***	2.98***	2.44***
	(0.79 - 1.85)	(1.41 - 1.90)	(-3.80 - 1.52)	(1.57 - 1.61)	(1.56 - 3.01)	(1.75 - 2.32)	(2.70 - 3.26)	(2.41 - 2.48)
Secondary	2.24***	3.64***	4.47***	2.73***	3.08***	3.14***	3.55***	3.41***
	(1.65 - 2.82)	(3.19 - 4.10)	(2.58 - 6.37)	(2.70 - 2.75)	(2.28 - 3.88)	(2.79 - 3.49)	(3.44 - 3.65)	(3.36 - 3.46)
Tertiary	2.76***	4.93***	1.96**	2.91***	5.58***	9.36***	6.19***	5.92***
	(1.97 - 3.55)	(2.60 - 7.25)	(0.40 - 3.52)	(2.85 - 2.97)	(4.51 - 6.65)	(8.28 - 10.44)	(5.99 - 6.39)	(5.86 - 5.99)
**HH consumption per capita (ref:Q1)**								
Q2	-0.28	0.02	-0.07	-0.55***	-0.15	0.69***	0.57***	0.01
	(-0.77 - 0.20)	(-0.59 - 0.62)	(-0.79 - 0.65)	(-0.57 - -0.52)	(-0.78 - 0.48)	(0.27 - 1.10)	(0.27 - 0.86)	(-0.07 - 0.09)
Q3	-0.42*	0.35	-0.75	-0.54***	0.23	-0.25	0.43***	0.46***
	(-0.91 - 0.08)	(-0.18 - 0.88)	(-1.71 - 0.21)	(-0.59 - -0.49)	(-0.42 - 0.87)	(-0.81 - 0.31)	(0.36 - 0.49)	(0.35 - 0.56)
Q4 (richest)	-0.65**	-0.34***	-0.46***	-0.14***	0.83**	1.64***	2.23***	1.79***
	(-1.17 - -0.13)	(-0.59 - -0.09)	(-0.61 - -0.31)	(-0.16 - -0.12)	(0.15 - 1.51)	(1.48 - 1.81)	(1.98 - 2.49)	(1.72 - 1.86)
**Work type**								
Self-employed	-0.06	0.63	-2.54***	0.15***	0.72	2.05***	-2.22**	0.97***
	(-0.92 - 0.81)	(-0.12 - 1.38)	(-3.85 - -1.24)	(0.14 - 0.16)	(-0.41 - 1.85)	(1.18 - 2.92)	(-4.06 - -0.37)	(0.78 - 1.16)
Farming	-0.81**	0.80	-0.82***	-0.88***	-0.63	1.59**	-1.06***	-0.80***
	(-1.43 - -0.19)	(-0.19 - 1.78)	(-1.23 - -0.40)	(-0.90 - -0.85)	(-1.44 - 0.18)	(0.33 - 2.85)	(-1.17 - -0.94)	(-0.96 - -0.64)
Unemployed/retired	-4.18***	-0.42	-2.04***	-3.22***	0.17	3.69***	0.52***	1.21***
	(-4.85 - -3.52)	(-1.83 - 0.99)	(-2.81 - -1.28)	(-3.28 - -3.17)	(-0.71 - 1.04)	(2.73 - 4.65)	(0.17 - 0.86)	(1.05 - 1.36)
**Functional limitation**								
Reported functional limitation	-13.83***	-13.89***	-13.44***	-16.22***	-7.84***	-5.83***	-7.28***	-9.31***
	(-14.31 - -13.35)	(-16.49 - -11.29)	(-15.78 - -11.11)	(-16.25 - -16.19)	(-8.47 - -7.21)	(-7.03 - -4.63)	(-8.52 - -6.04)	(-9.34 - -9.27)

Note: All estimates adjusted with sample weight
a: Coefficients were estimated using multilevel mixed-effect model
b: Coefficients were estimated using quantile regression analysis

Figure S1 Variables used to proxy SF-36 in CHARLS, and comparing with items from standard SF-36 instrument

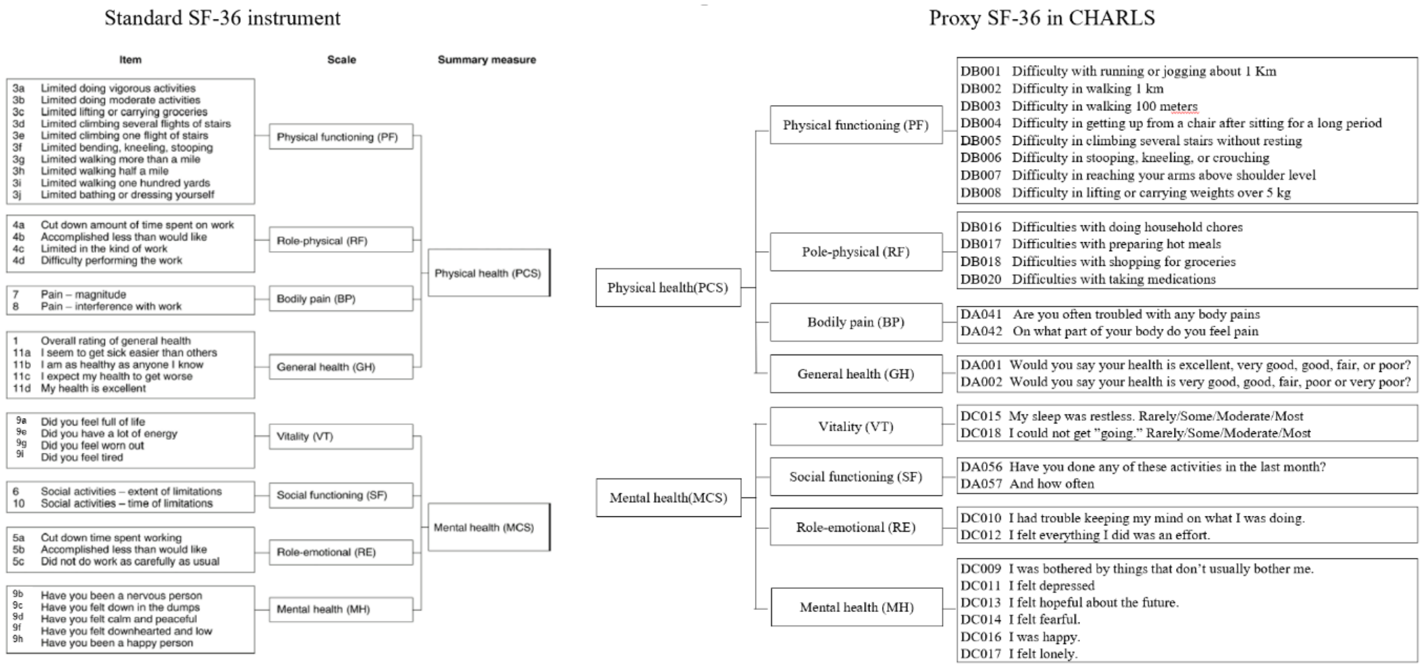

Figure S2. Estimated mean Physical Component Scores (PCS) and Mental Component Scores (MCS) scores by age, education level and residency

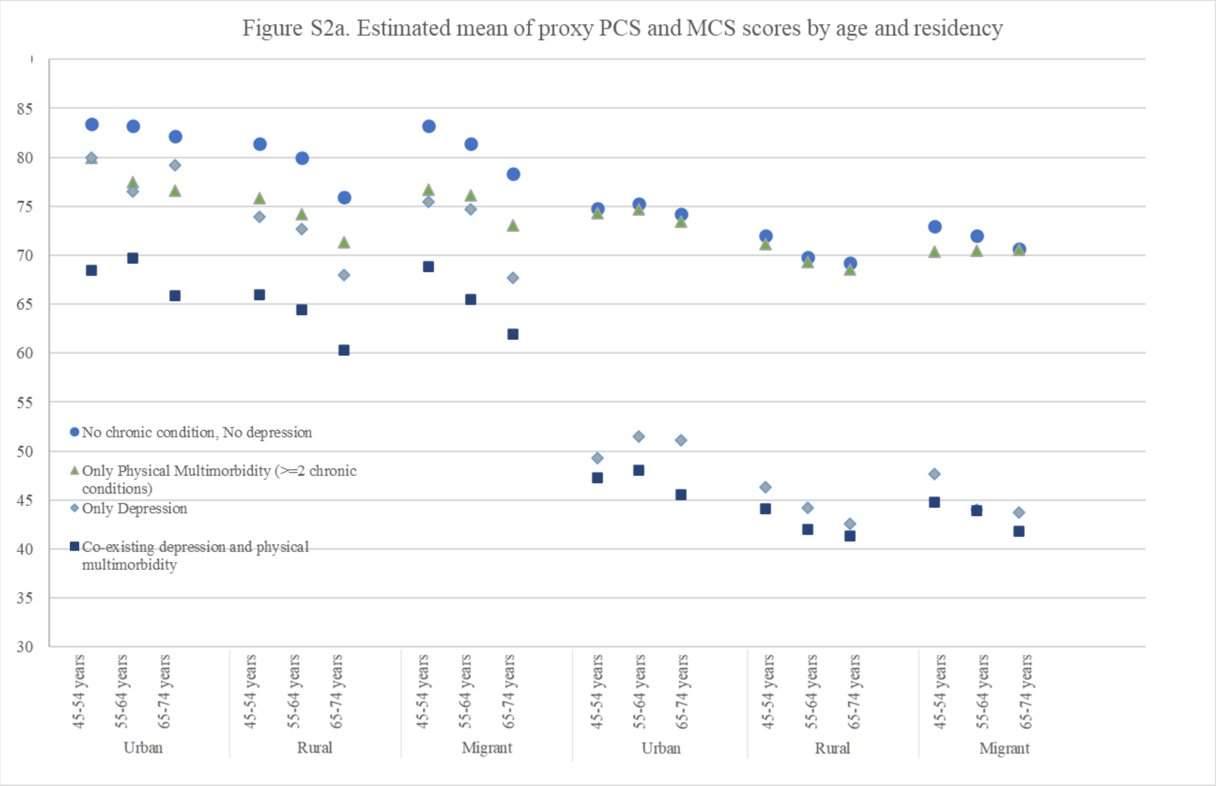

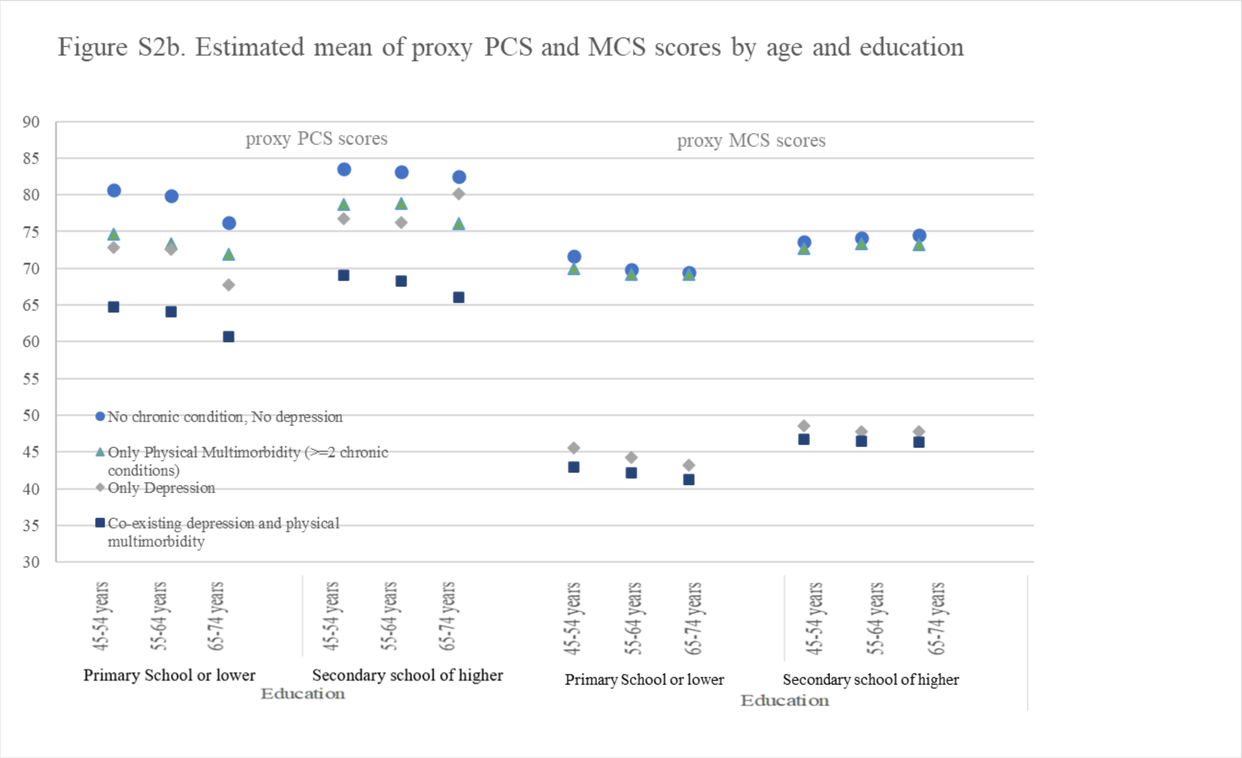

Fig S3. Estimated mean proxy Physical Component Scores (PCS) and Mental Component Scores (MCS) scores by multimorbidity burden and by age groups and gender among adults aged 45 and older

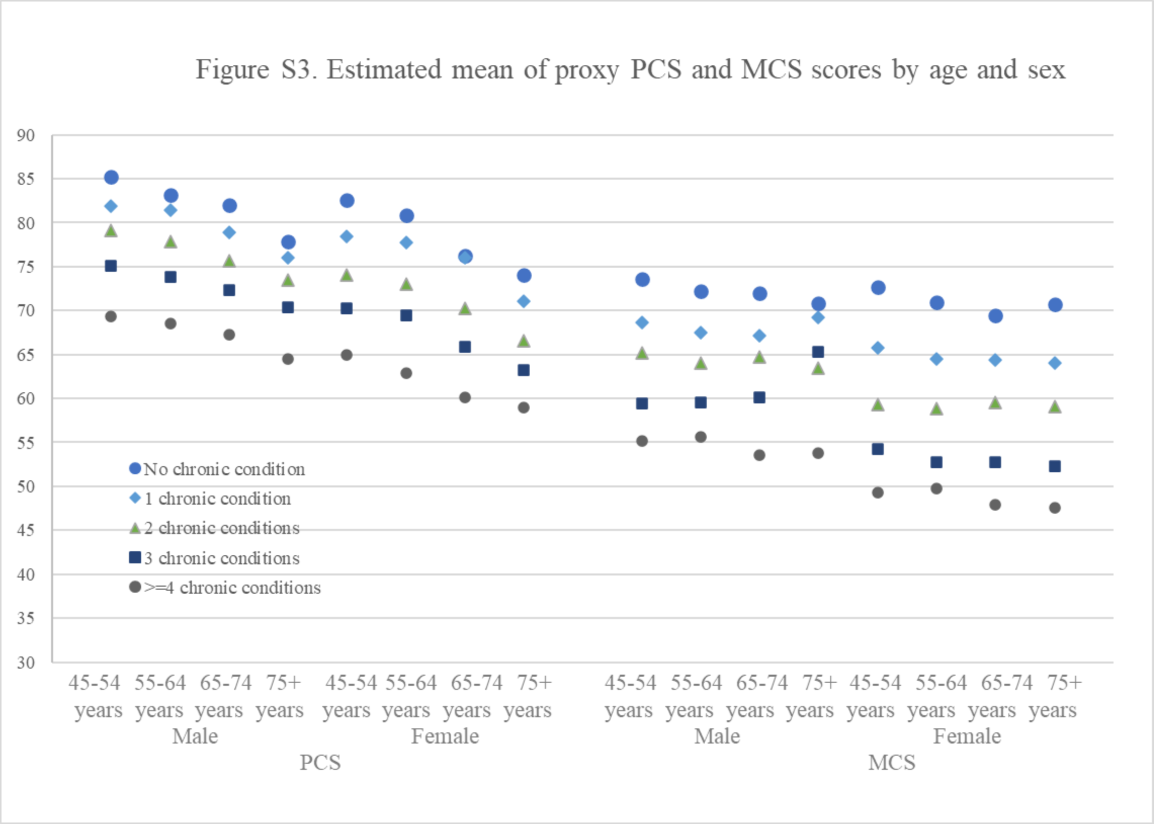

Supplement: Supplementary file 1 — Supplementary Information. [file 41598_2022_25092_MOESM1_ESM.pdf]
